# The foot-health of people with diabetes in a regional Australian population: a prospective clinical audit

**DOI:** 10.1186/1757-1146-5-6

**Published:** 2012-03-08

**Authors:** Byron M Perrin, Marcus J Gardner, Susan R Kennett

**Affiliations:** 1 La Trobe Rural Health School, La Trobe, University, Bendigo 3552, Australia; 2 Outpatient Rehabilitation Services, Bendigo Health, PO Box 126, Bendigo 3552, Australia; 3 Musculoskeletal Research Centre, La Trobe University, Bundoora 3086, Australia; 4 Bendigo Community Health Services, Bendigo 3552, Australia

**Keywords:** Podiatry, Diabetic Foot, Epidemiology, Rural Health

## Abstract

**Background:**

There is limited understanding of the foot-health of people with diabetes in Australian regional areas. The aim of this study was to document the foot-health of people with diabetes who attend publically funded podiatric services in a regional Australian population.

**Methods:**

A three month prospective clinical audit was undertaken by the publically-funded podiatric services of a large regional area of Victoria, Australia. The primary variables of interest were the University of Texas (UT) diabetic foot risk classification of each patient and the incidence of new foot ulceration during the study period. Age, gender, diabetes type, duration of diabetes and the podiatric service the patients attended were the other variables of interest.

**Results:**

Five hundred and seventy six patients were seen during the three month period. Over 49% had a UT risk classification at a level at least peripheral neuropathy or more serious diabetes-related foot morbidity. Higher risk at baseline was associated with longer duration of diabetes (F = 31.7, *p *< 0.001), male gender (*χ*^2 ^= 40.3, *p <*0.001) and type 1 diabetes (*χ*^2 ^= 37.3, *p <*0.001). A prior history of foot pathology was the overwhelming predictor for incident ulceration during the time period (OR 8.1 (95% CI 3.6 to 18.2), *p *< 0.001).

**Conclusions:**

The publically funded podiatric services of this large regional area of Australia deal with a disproportionally large number of people with diabetes at high risk of future diabetes-related foot complications. These findings may be useful in ensuring appropriate allocation of resources for future public health services involved in diabetic foot health service delivery in regional areas.

## Background

Diabetes-related foot complications pose a significant burden to health care systems and can be devastating to an individual [1]. People with diabetes can develop complications such as peripheral neuropathy, skin ulcerations on the feet and lower limb amputations [2]. Other complications due to diabetes can include Charcot neuropathic osteoarthropathy [3] and peripheral arterial disease [4]. It is estimated that diabetes-related foot ulceration resulted in nearly 10,000 Australian hospital admissions for the year 2004-2005 [5], and the number of diabetes-related lower-limb amputations performed in Australia has increased from approximately 2,600 each year for the years 1995-1998 [6] to 3,400 during 2004-2005 [5]. Diabetes-related foot complications also have significant deleterious effect on quality of life [7] and recent Australian research indicates these complications may be disproportionately found in socially disadvantaged populations [8].

Almost without exception peripheral neuropathy has been shown to be an independent risk factor for future ulceration [9], and its deleterious effect on the protective sensation of the feet of a person with diabetes to protect their feet from injury and trauma is well documented [10]. Over ten years ago, the population-based, cross-sectional Australian Diabetes, Obesity, and Lifestyle Study (AusDiab) found that 10% of people with diabetes in Australia showed signs of peripheral neuropathy, with 2.1% reporting a history of diabetes-related foot ulceration [11,12]. An Australian study of participants enrolled from a tertiary level metropolitan diabetes centre reported a prevalence of 17% of people with diabetes with peripheral neuropathy [13], and the National Association of Diabetes Centres reports a prevalence of peripheral neuropathy in Australian diabetes health centres of 24% [14]. These higher figures are comparable to a large study from the United Kingdom, which reported a prevalence of peripheral neuropathy in a clinical population of close to 20% [15]. There is limited other Australian data that describes a large sample of people with diabetes with respect to various foot-health characteristics, especially within rural or regional settings.

**Figure 1:**
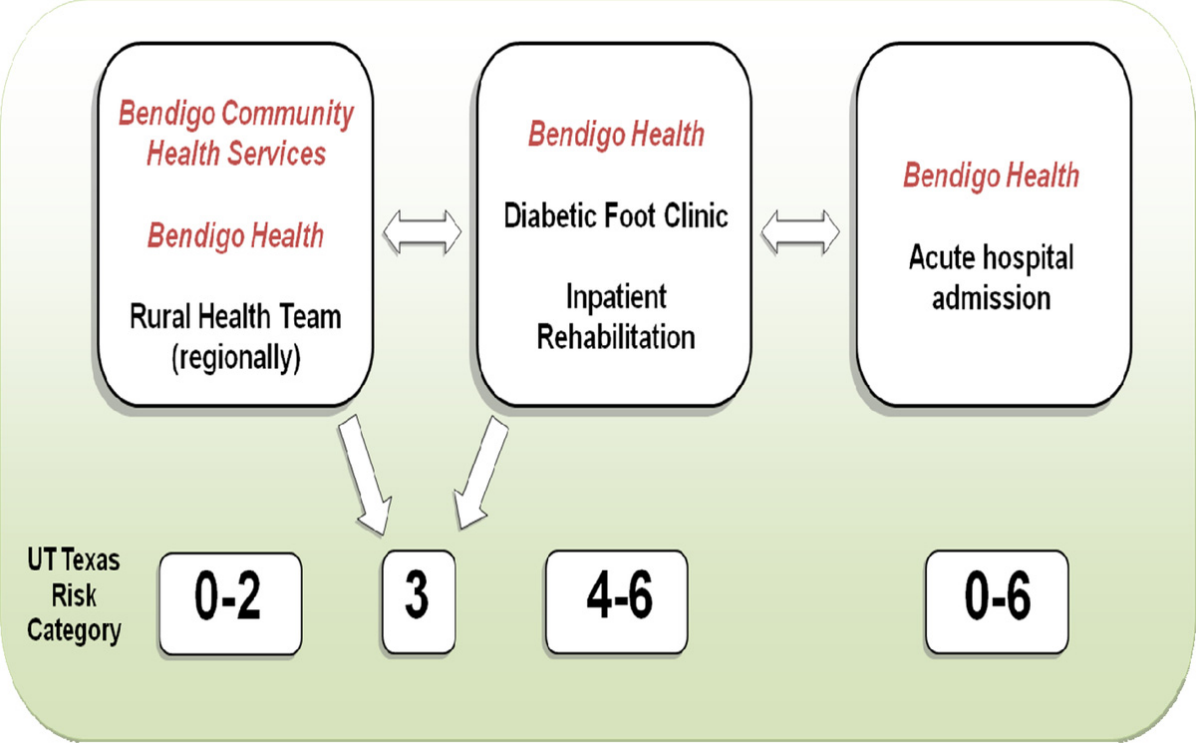
**The Podiatry Diabetes Model**. The Podiatry Diabetes Model aims to ensure that a patient with diabetes is seen by the most appropriate podiatric service of Bendigo Health and Bendigo Community Health Services according to the University of Texas Risk Classification [16].

This study reports on the activities of the publically-funded podiatry services within the Greater Bendigo area of the Loddon Mallee region of Victoria, Australia. The publically-funded podiatry clinics are delivered by a large regional hospital (Bendigo Health) and a community health service (Bendigo Community Health Services). In 2010, there were 8.6 full-time equivalent publically-funded podiatrists providing services to people with diabetes in the region, acting across the two organisations and within multiple podiatric services (Table 1). Eligibility criteria for the podiatric services were consistent with the aims of the specific funding source for each service, which ranged from helping maintain independence in frail-aged and disabled populations (the Home and Community Care program) to preventing re-admission for serious diabetes-related foot complications (the Hospital Admissions Risk Program). In addition to the services provided within Bendigo itself (a regional city of approximately 100,000 people) outreach clinics are also conducted in a range of small and relatively isolated rural towns. These podiatric services involve a catchment area of approximately 200,000 people. All the podiatric services at the two organisations approach the care of people with diabetes using an established "Podiatry Diabetes Model" (Figure 1) [16]. Within the model, the multiple podiatric services of Bendigo Health and Bendigo Community Health Services are grouped into three categories: community, sub-acute and acute (Table 1). The fundamental goal of the model is to help direct a patient with diabetes to the most appropriate podiatric service equipped to manage that patient's future risk of diabetes-related foot complications as determined by an established risk classification tool. The model has been previously evaluated [16] and is effective in achieving this goal.

**Table 1:** The podiatric services involved in this study

Organisation	Podiatric Service	Source of Funding	Aims of Funding	Podiatry FTE	Service category within model
Bendigo Services	Community Health	Home and Community Care (HACC)	Frail-aged and disabled people. Example case: an older or disabled person who is living independently in the community	3.7	Community
			
		Early Intervention Chronic Disease (EICDM)	Early intervention for management of chronic disease	0.6	
			
		Enhanced Primary Care (EPC)	Medicare funded initiative that includes allied health services to enhance preventative health care for people with chronic conditions. Example case: a person with diabetes living independently in the community	0.4	
			
Bendigo Health	Rural Health Team	Home and Community Care (HACC)	Frail-aged and disabled people	2.8	
			
		Rural Primary Health Service (RPHS)	Integrated primary care for chronic disease in rural and remote areas. Example case: a person with diabetes residing in a rural area0.2		
		
	Diabetic Foot Clinic	Victorian Ambulatory Classification System (VACS)	Prevention of readmission to hospital for known co morbidity. Example case: a person with a previous admission to hospital due to a diabetes-related foot problem	0.4	Sub-acute
			
	Inpatient Rehabilitation	Casemix Rehabilitation and Funding Tree (CRAFT)	Provide rehabilitation post. Lower limb amputation to optimise patient function and independence. Example case: a person with diabetes undertaking inpatient rehabilitation inpatient following a lower limb amputation	0.1	
	
	Acute hospital	Hospital Admissions Risk Program (HARP)	Reduce risk of re-hospitalisation/emergency department presentation in people with chronic diseases and complex needs. Example case: a person currently admitted to hospital with a diabetes- related foot problem	0.2	Acute
			
	Acute hospital	Geriatric Evaluation and Management (GEM)	Inpatient care of chronic conditions associated with aging, cognitive dysfunction, chronic illness or loss of functional ability. Example case: an elderly person admitted to hospital who has diabetes	0.2	

The aim of this study was to document basic diabetes-related foot-health characteristics of the patients who attended the diverse range of publically-funded podiatric services included in the Podiatry Diabetes Model.

## Methods

This project was approved by the Human Research Ethics Committee of Bendigo Health. It was a three month prospective clinical audit held in 2010 between March and May inclusive. Every podiatric consultation between a podiatrist from Bendigo Health and Bendigo Community Health Services with a patient with diabetes was recorded. Only data usually recorded by a podiatrist within the Podiatry Diabetes Model in a clinical consultation was recorded.

The primary variables of interest were the University of Texas (UT) diabetic foot risk classification [17,18] designated to each patient on the initial visit and the incidence of new diabetes-related foot ulceration during the three-month period. The UT risk classification system (Table 2) was chosen as it has been shown to be a reliable, valid and predictive tool for identifying future foot-health outcomes for people with diabetes [17,18]. All the podiatrists involved in the study were expected to make a judgement on future risk of ulceration or amputation based on assessment recommendations for the UT risk classification system. Secondary variables included the site at which the patient was seen for the initial visit (community, sub-acute or acute) and other basic demographic and medical variables that included age, gender, type of diabetes and duration of diabetes.

**Table 2:** The University of Texas risk classification system (adapted from [17])

0 No neuropathy	1 Peripheral neuropathy	2 Neuropathy with deformity	3 History of pathology
Sensation intact: 10 g monofilament detectable or vibratory perception threshold < 25 volts Vascular status intact: Ankle brachial index > 0.8, toe systolic pressure > 45 mmHg No history of neuropathic ulceration or Charcot neuropathic osteoarthropathy. Foot deformity may be present	Loss of protective sensation: 10 g monofilament not detectable or vibratory perception threshold > 25 volts Vascular status intact No history of neuropathic ulceration or Charcot neuropathic osteoarthropathy. No foot deformity	Loss of protective sensation Vascular status intact No history of neuropathic ulceration or Charcot neuropathic osteoarthropathy. Foot deformity present	Loss of protective sensation Vascular status intact History of neuropathic ulceration or Charcot neuropathic osteoarthropathy
**4A** **Neuropathic ulceration**	**4B** **Acute Charcot arthropathy**	**5** **Infected foot**	**6** **Ischaemia**

Loss of protective sensation Vascular status intact Non-infected neuropathic ulceration No acute Charcot neuropathic osteoarthropathy.	Loss of protective sensation Vascular status intact Non-infected neuropathic ulceration may be present Acute Charcot neuropathic osteoarthropathy present	Loss of protective sensation Vascular status intact Infected wound Charcot neuropathic osteoarthropathy may be present	Sensation may or may not be intact Ankle brachial Index < 0.8 or toe systolic pressure < 45 mmHg Ulceration may be present

For the statistical analysis, the number of risk categories was consolidated. The University of Texas risk classification system has eight risk categories in total as shown in Table 2. For statistical analysis this was reduced to four: no neuropathy, neuropathy, history of pathology and active foot pathology. The UT risk classification "neuropathy with deformity" was pooled with the "peripheral neuropathy" category and all University of Texas risk classifications that described a current, active diabetes-related foot complication ("neuropathic wound", "acute Charcot Arthropathy", "infected foot", "ischaemic foot") were pooled into a new "active foot pathology" category. Whilst the pooled categories deviate from the eight category UT Texas risk classification system, the categories are still ordered in a logical clinical fashion to reflect increasing risk of diabetes-related foot ulceration and lower limb amputation.

Basic participant characteristic data were collected and summarised using means and standard deviations for continuous data. Standard chi-square test for independence was used to examine the relationship between variables with categorical data and one-way between groups analysis of variance with post hoc tests was used to examine the relationship between variables with continuous data. Multivariate logistic regression was used to determine the independent risk factors for incident ulceration during the three-month period. Variables statistically significantly associated with incident ulceration after separate bivariate analyses were included in the logistic regression modelling.

## Results

Five hundred and seventy-six patients with diabetes were seen during the study period, and basic participant characteristics can be found in Table 3. The participants were older, had a slight preponderance of males, overwhelmingly had type 2 diabetes and a mean duration of diabetes of over ten years. Just over fifty percent of the sample had a UT risk classification of "no neuropathy", with 25.0% classified as "neuropathy" or "neuropathy and deformity", 13.0% classified as "history of pathology" and a total of 10.6% classified as having an active diabetes-related foot problem (Table 4).

**Table 3:** Participant characteristics

Variable	Total (n = 576)
Age (years)	71.3 ± 11.6

Male gender (%)	53.3

Type 2 diabetes (%)	95.8

Duration of diabetes (years)	12.1 ± 10.0

Participants at each site at baseline (%)	85.6
Community	11.6
Subacute	2.8
Acute	

**Table 4:** Number of patients seen per UT risk category at baseline

Pooled risk category	Frequency (%)
No Neuropathy	296 (51.4)

Neuropathy	144 (25.0)

History of Pathology	75 (13.0)

Active pathology	61 (10.6)

Total	576 (100.0)

There was a statistically significant difference between the risk categories according to age (F = 11.9, *p *< 0.001). Those classified as having "neuropathy" (75.5 ± 9.5) were older than those classified as "no neuropathy" (70.7 ± 11.7), "history of pathology" (69.3 ± 11.6) and "active pathology" (66.2 ± 11.6). Those classified as having an active pathology were also significantly younger than those with no neuropathy (*p *= 0.02). There was a statistically significant difference between the risk categories according to duration of diabetes (F = 31.7, *p *< 0.001), which showed a linear trend for increased duration of diabetes and higher risk. Those classified as having "no neuropathy" (9.1 ± 7.6) had a significantly shorter duration of diabetes than those classified as "neuropathy" (12.3 ± 8.6), "history of pathology" (17.9 ± 12.2) and "active pathology" (19.1 ± 13.3). Those with a history of pathology (*p <*0.001) or an active pathology (*p *< 0.001) had significantly longer duration of diabetes than those with neuropathy. There was a clear pattern of males being more associated with higher risk (*χ*^2 ^= 40.3, *p *< 0.001). There was also a clear pattern of type 1 diabetes being associated with higher risk at baseline (*χ*^2 ^= 37.3, *p *< 0.001).

**Figure 2:**
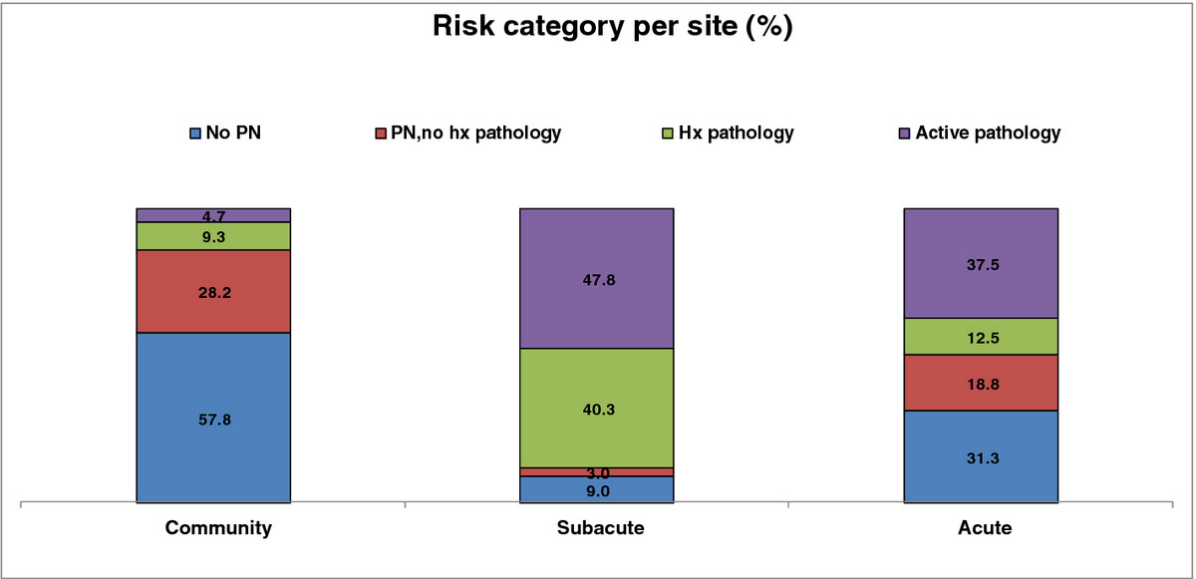
**The proportion of patients seen at baseline at each site according to risk category**.

The community, sub-acute and acute podiatric services each saw 493 (85.6%), 67 (11.6%) and 16 (2.8%) patients respectively. There were 919 total podiatric contacts across the three sites, with the community, sub-acute and acute sites accounting for 634 (69.0%), 226 (24.6%) and 59 (6.4%) of the contacts respectively. The acute (2.7 ± 4.2) and sub-acute (3.1 ± 2.3) services had significantly more contacts during the study period per person than the community service (1.3 ± 0.6, F = 79.4, *p *< 0.001). Detailed analysis of the proportions of participants classified in each risk category that were seen at each of the community, sub-acute and acute sites have been published elsewhere [16]. A summary is shown in Figure 2, which shows a much higher proportion of patients at lower risk of diabetes-related foot complications seen at the community podiatry services, and a much higher proportion of patients at higher risk of future diabetes-related foot problems seen on the sub-acute services. There was more of a mixture of patients seen on the acute services.

Thirty-six (6.3%) people developed a new, incident diabetes-related foot ulceration during the study period (Table 5). Separate bivariate analysis showed that patients who developed ulceration during the three-month period were younger (t = 3.5, *p *= 0.001) and had a longer duration of diabetes (t = -3.3, *p *= 0.002). The proportion of patients with type 1 diabetes who developed incident ulceration was higher than for patients with type 2 diabetes (*χ*^2 ^= 9.1, *p *= 0.003). The proportion of patients with a history of diabetes-related pathology who developed incident ulceration was much higher than the proportion of patients who did not have a history of pathology (*χ*^2 ^= 54.2, *p *< 0.001).

**Table 5:** Bivariate analysis of incident ulceration

Variable	Ulceration (n = 36)	No ulceration (n = 540)	Magnitude of difference	*p*
Diabetes duration (years)	18.6 ± 12.5	11.6 ± 9.7	Cohen's d = 0.7 MD 7.0 (2.7 to 11.3)	0.002*

Age (years)	64.8 ± 11.4	71.7 ± 11.5	Cohen's d = 0.6 MD -6.9 (-2.9 to -10.8)	0.001*

Past foot pathology (%)	Yes: 20.0 No: 2.2	Yes: 80.0 No: 97.8	Cramer's V = 0.3 OR 10.9 (5.1 to 23.3)	< 0.001*

Diabetes type (%)	1: 20.8 2: 5.6	1: 79.2 2: 94.4	Cramer's V = 0.1 OR 4.4 (1.55 to 12.6)	0.003*

Gender (%)	M: 7.2 F: 5.2	M: 92.8 F: 94.8	Cramer's V = 0.1 OR 0.04 (0.7 to 2.8)	0.330

Bivariate relationships were analysed used Chi-square analysis for categorical data and independent samples *t*-test for continuous data. OR = unadjusted odds ratio (95% confidence interval). MD = mean difference (95% confidence interval)

*Statistically significant

Stepwise logistic regression analysis with variables entered in order according to their bivariate effect size (from highest to lowest) showed only prior history of diabetes-related foot problem and younger age remained as risk factors for incident ulceration (Table 6). The Hosmer and Lemeshow Test confirmed the model was a good fit (*χ*^2 ^= 9.9, *p *> 0.05) and the Nagelkerke R Square test indicates that the four variables that were significantly associated with incident ulceration after the bivariate analyses accounted for up to 23.4% of the variance for the logistic regression model, of which history of pathology accounted for over 18.0% of the variance.

**Table 6:** Logistic regression analysis of incidence of pathology as a function of significant variables after separate bivariate analyses

Variables	β	Wald test	OR (95% CI)	*p*
**History of pathology**	2.1	25.3	8.1 (3.6 to 18.2)	< 0.001*

**Age**	-0.04	6.9	0.96 (0.93 to 1.0)	0.010*

**Diabetes duration**	0.03	2.8	1.0 (1.0 to 1.1)	0.100

**Type 1 diabetes**	0.75	1.0	2.1 (0.5 to 9.2)	0.320

*Statistically significant

## Discussion

The study design used in this study was a prospective clinical audit, where accurate recording of socio-demographic and foot-health variables across populations of people with diabetes attending publically funded community, sub-acute and acute health-care podiatric services in a regional Australian area was undertaken. A standardised clinical approach to assessment, diagnosis and management of the care of people with diabetes was used that allowed accurate data to be recorded prospectively for a period of three months. This was facilitated by the creation and utilisation of the Podiatry Diabetes Model [16], that focuses on ensuring efficient use of the available podiatric services. Fundamental to this is the accurate diagnosis risk for future diabetes-related foot complications and the timely referral to the podiatric service best equipped to oversee an appropriate management plan. This podiatric model of care has been shown in a validation study to have been functioning successfully in this way [16], and the recommendations from the initial validation study of the model are being implemented. These include the future inclusion of other podiatric or health services (e.g. general medical practice) that are currently not included in the model to further broaden the sample population.

During the three month period, over five hundred and seventy-six patients with diabetes were seen by the podiatric services of Bendigo Health and Bendigo Community Health Services. Just under fifty percent of the sample had peripheral neuropathy or more serious diabetes-related foot morbidity, a higher prevalence found than in other clinical populations in Australia and the United Kingdom [13,15]. Over twenty three percent of the patients either had a serious active diabetes-related foot complication at baseline or had a history of one. The incidence of new serious foot complications during the three month period was high at six percent of the sample. These figures suggest that a high proportion of patients with diabetes seen by the public podiatry services of the Greater Bendigo area of the Loddon Mallee region had generally poor foot health.

This is consistent with recent research that has shown that the Loddon Mallee region of Victoria (which is in the catchment areas for the Podiatry Diabetes Model) has some of the highest rates hospital separations related to diabetes-related foot complications in the state of Victoria [8]. Other research of a sample of over one hundred people with diabetes in the region who attended the Diabetic Foot Clinic (the subacute podiatric service of the Podiatry Diabetes Model which focuses its service to people at UT risk category 3 or above) of Bendigo Health showed an annual incidence of diabetes-related foot ulceration of over thirty percent, a very high figure [19].

Basic demographic and diabetes-related information gathered at baseline in this study further enhances the understanding of this high-risk population. There were generally an even proportion of males and females in the sample, with a slight preponderance of males. The distribution of ages for the patients in the sample suggested the majority of the sample were over the age of sixty, with a mean age of just over seventy years. Again, this is consistent with data for the Loddon Mallee region, which has an older population than Australia as a whole and a higher proportion of people aged over fifty-five years [20]. Consistent with other studies, the patients with peripheral neuropathy were found to be significantly older than those without peripheral neuropathy [12]. However, less common are the findings where patients with a current diabetes-related foot complication were significantly younger than those without peripheral neuropathy. A plausible explanation is that those with an active foot complication at baseline had a significantly longer duration of diabetes, which is consistent with findings from the AusDiab population-based study, where duration of diabetes (in addition to older age) was associated with peripheral neuropathy [12].

Although not collected in this study, it is possible that socioeconomic variables may be related to the foot health of people with diabetes in a regional population. Bergin and colleagues [8] analysed diabetes-related hospital separations across some of the most advantaged and the least advantaged regions in the state of Victoria by using the Index for Relative Socio-economic Disadvantage as measured by the Australian Bureau of statistics [21]. The Index for Relative Socio-economic Disadvantage provides a general measure of disadvantage by using indicators of low socio-economic well-being as measured by each census to determine a summary index, and to indicate the proportion of relatively disadvantaged people within a particular area [21]. Data from the 2006 Australian population census has shown that six of the nine regions of the Loddon-Mallee are more disadvantaged than 70% of the other regions in Victoria [22,23]. A recent review of global incidence rates of diabetes-related lower limb amputations has also found that social deprivation may be significant [24]. Future research should investigate the specific relationship between socio-economic disadvantage and diabetes-related foot health.

The incidence of new ulceration during the three-month period was over six percent. Separate bivariate analyses of the demographic and diabetes-related variables measured show strong associations with new incident ulceration with younger age, longer duration of diabetes, type 1 diabetes and a prior history of diabetes-related foot pathology. After the unadjusted analysis, both longer duration of diabetes and younger age had a strong relationship with new incidence of ulceration. However, when history of pathology was added to the logistic regression multivariate analysis longer duration of diabetes became a non-significant predictor of incident ulceration, and the effect of age was reduced from a medium effect to a low effect. This suggests that there was confounding between duration of diabetes and age with history of pathology. When the effects of the confounding were removed, history of pathology remained the most significant risk factor for new incident ulceration, with an odds ratio indicating those with a history of pathology were eight times more likely to present with a new diabetes-related foot ulceration during the time period than those who did not have a history of pathology. This finding is consistent with those found by the developers of the UT risk classification system, who found a thirty-six fold cumulative increase in risk of ulceration for those with a history of pathology in their sample [18].

The results of this study show that a surprisingly high number of patients were seen at the community podiatric services of the region that were designated as higher risk, including having peripheral neuropathy or a history of pathology. This may reflect the contextual influences on the regional publically funded podiatry services that include adherence to service funding requirements and the high demand for subsidised podiatry in the region. Further population-based research is required that is inclusive of the private podiatry services in the region to better understand if this high proportion of high-risk patients exists in other areas of the regional community of people with diabetes. For the community based podiatric services in this study, access to the Diabetic Foot Clinic is possible as required, however this may not be the case for many other regional areas of Australia. It is important to recognise that regional publically funded podiatric services may be managing large numbers of patients at high risk of diabetes-related foot complications and steps should be made to ensure podiatry staff in these clinics are well supported to provide the best possible care for these patients. Important to this could be the utilisation of standardised clinical guidelines, particularly as Australian research has shown that in community podiatry settings clinical guidelines are under-utilised [25]. The Podiatry Diabetes Model uses the UT Texas risk classification to guide assessment and diagnosis for the community, sub-acute and acute podiatry settings, and there have been recent comprehensive national Australian guidelines produced to aid health clinicians in the prevention, identification and management of diabetes-related foot complications [1].

Although there are a high proportion of patients in this broad clinical sample who are at high risk of future foot problems, there are still a large number of patients who are at lower risk of serious foot complications such as ulceration, Charcot neuropathic osteo-arthropathy or peripheral arterial disease. There is a large amount of low level evidence to suggest that podiatric care and basic preventative foot care behaviours can be useful in this population and the American Diabetes Association recommend basic preventative foot care activities be undertaken such as regular monitoring of the feet, appropriate care of the skin and nails and the use of appropriate footwear [26]. The podiatrists working within the Podiatry Diabetes Model work within these guidelines. However, a Cochrane review suggests that patient education for the prevention of diabetes-related foot complications is yet to be proven to be effective, with education possibly having positive outcomes on foot-care behaviours in the short term only, with a yet unknown effect on long term foot-health outcomes [27]. In line with the monitoring and preventative programs required for patients at high risk, the few reported successful education programs for those at lower risk are also labour intensive and require adequate resourcing [28].

The results found in this study must be considered in the context of the limitations of the study design. The duration of the study period was relatively short at three months only. Although this period allowed for a large sample of patients to be considered in the study for the cross-sectional analysis, the data related to risk factors for new, incident ulceration may have been strengthened with at least a six month or preferably one year follow-up period. Even though the data collected in this study clearly shows a high incidence of ulceration in the sample, it is difficult to compare to other studies that generally have a much longer follow-up time. It is intended that a future study over a longer period of time will commence soon in the same region to overcome this issue. Caution is recommended in comparing the results of this study to other similar clinical population studies from Australia [13,14] and the United Kingdom [15] (see above). Whilst the participants in this sample are regarded as coming from a clinical population a large proportion of the sample attended the podiatric service within PDM that sees patients for primary prevention of diabetes-related foot complications in a community setting.

The UT risk classification categories as originally determined in this study were pooled into a fewer number of risk categories based on established risk factors for diabetes-related foot complications. Whilst this strengthened the statistical analysis, this may detract from comparing the results of the study to other studies that report the use of the UT risk classification system without pooling the risk categories. Caution should also be taken in comparing the results of this study with other studies that utilised other risk classification tools available that integrate risk factors (such is ischaemia) to designate risk categories differently to the UT Texas system [29]. Furthermore, although a standardised approach to classifying risk of future diabetes-related foot problems was used across all the podiatric services within the Podiatry Diabetes Model, it is possible that there may be misclassification or differences in interpretation of the UT risk classification system across the multiple podiatrists involved in collecting data. This may be particularly relevant for the diagnosis of "ischaemia" as an active pathology. Individual podiatrists anecdotally report a large number of instances of suspected falsely elevated ankle brachial index results, and not all the podiatrists had access to toe pressure plethysmography technology. This may have eventuated in an underestimation of people classified as having an ischaemic limb as designated by the UT risk classification system, or an "active pathology" in this study.

## Conclusions

The results of this study show that over half of all patients with diabetes attending the publically funded podiatric services of a large regional Australian area are at significant risk of future diabetes-related foot complications. This has potential implications for the future delivery of health services focusing on the foot health of people with diabetes in regional Australia.

## Abbreviations

UT: University of Texas; AusDiab: Australian Diabetes: Obesity and Lifestyle Study.

## Competing interests

The authors declare that they have no competing interests.

## Authors' contributions

BMP contributed to the conception, design, data collection, statistical analysis, interpretation and preparation of the manuscript. MJG contributed to the conception, design, data collection, interpretation and preparation of the manuscript. SRK contributed to the conception, design, data collection, interpretation and preparation of the manuscript. All authors read and approved the final manuscript
